# Pseudorabies Virus US3 Protein Inhibits IFN-β Production by Interacting With IRF3 to Block Its Activation

**DOI:** 10.3389/fmicb.2021.761282

**Published:** 2021-10-22

**Authors:** Jingying Xie, Xiangbo Zhang, Lei Chen, Yingjie Bi, Adi Idris, Shujuan Xu, Xiangrong Li, Yong Zhang, Ruofei Feng

**Affiliations:** ^1^Key Laboratory of Biotechnology and Bioengineering of State Ethnic Affairs Commission, Biomedical Research Center, Northwest Minzu University, Lanzhou, China; ^2^College of Veterinary Medicine, Gansu Agricultural University, Lanzhou, China; ^3^Menzies Health Institute Queensland, School of Pharmacy and Medical Science, Griffith University, Southport, QLD, Australia

**Keywords:** pseudorabies virus, US3 protein, innate immunity, IRF3, IFN-β

## Abstract

Pseudorabies virus is a typical swine alphaherpesvirus, which can cause obvious neurological disorders and reproductive failure in pigs. It is capable of evading host antiviral immune response. However, the mechanism by which many PRV proteins assist the virus to evade innate immunity is not fully understood. This study identified PRV US3 protein as a crucial antagonistic viral factor that represses interferon beta (IFN-β) expression. A in-depth study showed that US3 protein restricted type I IFN production by targeting interferon regulatory factor 3 (IRF3), a key molecule required for type I IFN induction. Additionally, US3 protein interacted with IRF3, degraded its protein expression to block the phosphorylation of IRF3. These findings suggested a novel strategy utilized by PRV to inhibit IFN-β production and escape the host innate immunity.

## Introduction

During a virus infection, host cellular recognition receptors (PRRs) recognize pathogen-associated molecular patterns (PAMPs) and trigger the induction of type I interferons (IFNs) and pro-inflammatory cytokines to restrict viral replication, clear up of infected cells, and further orchestrate the adaptive immune response to eradicate infected pathogens (Kawai and Akira, 2006; Carpenter et al., 2014; Beachboard and Horner, 2016; Chen et al., 2017). Among the PRRs, Cyclic GMP-AMP (cGAMP) synthase (cGAS) is a cytosolic DNA sensor and, when triggered, mounts a type I IFN response. Upon sensing pathogen DNA, cGAS catalyzes the synthesis of cGAMP, which activates the endoplasmic reticulum (ER)-anchored stimulator of interferon genes (STING). Stimulator of interferon genes then translocate from the ER to the Golgi apparatus to the recruit and phosphorylate TANK-binding kinase 1 (TBK1) and IκB kinase (IKK). These events then activate IRF3 and NF-κB to activate type I IFN production (Fitzgerald et al., 2003; Sharma et al., 2003; Sun et al., 2013; Xia et al., 2016).

To counteract the antiviral effects of cGAS-STING engagement, several DNA viruses, including Kaposi’s sarcoma-associated herpesvirus (Li et al., 2000; Ma et al., 2015; Wu et al., 2015), Herpes simplex virus 1 (Christensen et al., 2016; Su et al., 2016; Zhang et al., 2016; Zheng, 2018; Lin and Zheng, 2019; Zhu and Zheng, 2020), and Marek’s disease virus (Gao et al., 2019; Li et al., 2019), have evolved different evasion strategies. Pseudorabies virus (PRV), a member of the subfamily *Alphaherpesvirinae* of the family *Herpesviridae* (Mettenleiter, 2000), causes fatal fever and encephalomyelitis in pigs and susceptible animals (Sun et al., 2016). Although pigs are the natural host of PRV, other mammals, such as ruminants, carnivores, and rodents, are susceptible to PRV infection (Fonseca et al., 2010). Indeed, PRV infection is also known to cause human endophthalmitis in China (Ai et al., 2018; Fan et al., 2020; Liu et al., 2020; Wang et al., 2020). Importantly, PRV infection and the disease it causes have brought huge impact on economic for the swine industry.

Pseudorabies virus (PRV) is known to block type I IFN host antiviral responses. When PRV infects primary rat fibroblast cells, interferon-stimulated genes (ISGs) in these cells are suppressed (Brukman and Enquist, 2006b). The PRV glycoprotein gE/gI complex (Lamote et al., 2017), UL50 (Zhang et al., 2017), and EP0 (Brukman and Enquist, 2006a) can suppresses type I IFN host responses. Importantly, PRV UL13 inhibits cGAS-STING-mediated IFN-β production by phosphorylating IRF3 (Bo et al., 2020; Lv et al., 2020).

PRV protein kinase US3 has been shown to protect infected cells from apoptosis (Geenen et al., 2005; Qin et al., 2019). We reasoned that this could occur via exerting an uncharacterized antiviral evasion strategy and hypothesized that this could be occurring via the cGAS-STING pathway. US3 is a viral serine/threonine kinase, which is conserved in the alphaherpesvirus subfamily. Many studies indicated that Herpes simplex virus 1 (HSV-1) encoding US3 protein is involved in many processes during viral infection (Wagenaar et al., 1995; Leopardi et al., 1997; Reynolds et al., 2002; Cartier et al., 2003) and many other functions. Regarding how HSV-1 counteracts the host’s natural immune response, many studies showed that US3 could inhibit IFN-β (Wang et al., 2013; You et al., 2020) and Nuclear factor κB (NF-κB) (Wang et al., 2014) activation. Although HSV-1 US3 protein has multiple mechanisms for immune evasion, the immune evasion functions of PRV US3 are still poorly understood.

In this study, we found that PRV dampened IFN-β responses and that US3 protein impaired IFN-β production via degradation of IRF3. US3 also interacted with IRF3 and blocked its activation. Additionally, US3 knockdown partially recovered PRV infection-induced IRF3 degradation and IFN-β expression, suggesting PRV US3 could subvert antiviral innate immunity and evade host antiviral responses via a different mechanism compared to HSV-1 US3.

## Materials and Methods

### Cells and Viruses

The porcine kidney (PK15) cells were obtained from ATCC and cultured in DMEM supplemented with 10% new bovine serum (NBS) at 37°C in a 5% CO_2_ incubator. Pseudorabies Virus Bartha is an attenuated vaccine strain, obtained by extensive passaging of an Aujeszky strain isolated in Hungary (Christensen et al., 1992). Bartha-61 was propagated in BHK-21 cells, and the supernatants of infected cells were clarified and stored at −80°C.

### Antibodies and Reagents

Anti-FLAG tag rabbit polyclonal antibody (D110005), Anti-cGAS rabbit polyclonal antibody (D163570), HRP (horseradish peroxidase)-conjugated Goat Anti-Rabbit IgG (D110058) and HRP-conjugated Goat Anti-Mouse IgG (D110087) were purchased from Sangon Biotech (Shanghai, China). HA tag Polyclonal antibody (51064-2-AP) and IRF3 Polyclonal antibody (11312-1-AP) were purchased from Proteintech (Wuhan, China); STING (D2P2F) Rabbit mAb (13647S), Phospho-IRF-3 (Ser386) (E7J8G) XP^®^ Rabbit mAb antibody (37829S) and Myc-Tag (9B11) Mouse mAb (2276S) were bought from Cell Signaling Technology. Anti-HIST3H3 Polyclonal Antibody (K106623P) were purchased from Solarbio (Beijing, China). GAPDH Mouse Monoclonal Antibody (AF5009) and β-actin Mouse Monoclonal Antibody (AA128) were purchased from Beyotime Biotechnology (Shanghai, China). Anti-HSP90β antibody was purchased from Abbkine (ABP54794) (China).

*TransStart*^®^ Top Green qPCR SuperMix (+Dye II) was purchased from Transgen (Beijing, China). Cell membrane/cytoplasm/nuclear membrane protein step extraction kit (BB-31042) was bought from BestBio (Shanghai, China). Lipofectamine 3000 was purchased from invitrogen. Chemical reagents polybrene (Millipore), puromycin and RNase inhibitor (Thermo), MG132 (Beyotime), chloroquine (CQ) (tlrl-chq, InvivoGen) and ISD (tlrl-isdc, InvivoGen) were bought from indicated manufactures.

### Plasmids

Plasmids encoding HA-tagged cGAS and STING were constructed by molecular cloning methods. Myc tagged US3 plasmid was constructed in-house. All plasmids were verified by sequencing. The primer sequences used in this study are available upon request. pCMV-FLAG-TBK1, IRF3 constitutively active mutant IRF3/5D-FLAG and pCMV-FLAG-IRF3 expression plasmids were all constructed in-house.

### Western Blotting

Cells were harvested and whole-cell extracts were prepared with lysis buffer buffer RIPA (Solarbio, Beijing, China). Cell extracts were subjected to 10% or 15% SDS-PAGE, and the separated proteins were transferred to PVDF membranes (Millipore). The PVDF membranes were incubated with specific primary and HRP-conjugated secondary antibodies. GAPDH or β-actin was served as a loading control. The proteins were detected using ECL Blotting Substrates (Bio-Rad, CA, United States).

### Co-immunoprecipitation Assay

Cells were collected with a lysis buffer supplemented with a phosphatase inhibitor cocktail and incubated with anti-FLAG or anti-IRF3 antibody for 12 h at 4°. Then 10 μL of Protein G agarose slurry (Beyotime, China) was added to each lysate. After incubation for 4 h at 4°, the lysates were centrifuged at 2500 rpm for 5 min. The beads were collected and washed 5 times with ice-cold PBS. The precipitates were mixed with SDS buffer and boiled for 5 min at 95°. After centrifugation at 6000 rpm for 1 min, the supernatant was collected and used for western blot analysis.

### RNA Extraction and Real-Time Quantitative PCR

mRNA levels was determined for IFN-β using relative qPCR. Cellular RNA was isolated and reverse-transcribed to cDNA. Methods were performed as previously described (Xie et al., 2020). Primers for RT-qPCR are available upon request.

### CCK-8 Assay

Cell proliferation was determined using the CCK-8 assay. PK15 cells were seeded in 96-well plates overnight and then untreated or treated with MG132 or CQ. The proliferative ability of the cells was evaluated at 6 h and 12 h according to manufactures’ instruction.

### Statistical Analysis

Measurements were compared using a one-way ANOVA. Statistical significance comparisons were calculated using a Student’s *t*-test in GraphPad Prism 7.0 software (La Jolla, CA, United States). Values are expressed in graph bars as the mean ± SD of at least three independent experiments, unless otherwise noted. Asterisks denote statistically significant differences (^∗∗∗^ *p* < 0.001, ^∗∗^ *p* < 0.01, and ^∗^ *p* < 0.05).

## Results

### Pseudorabies Virus US3 Protein Blocks IFN-β Activation

**FIGURE 1:**
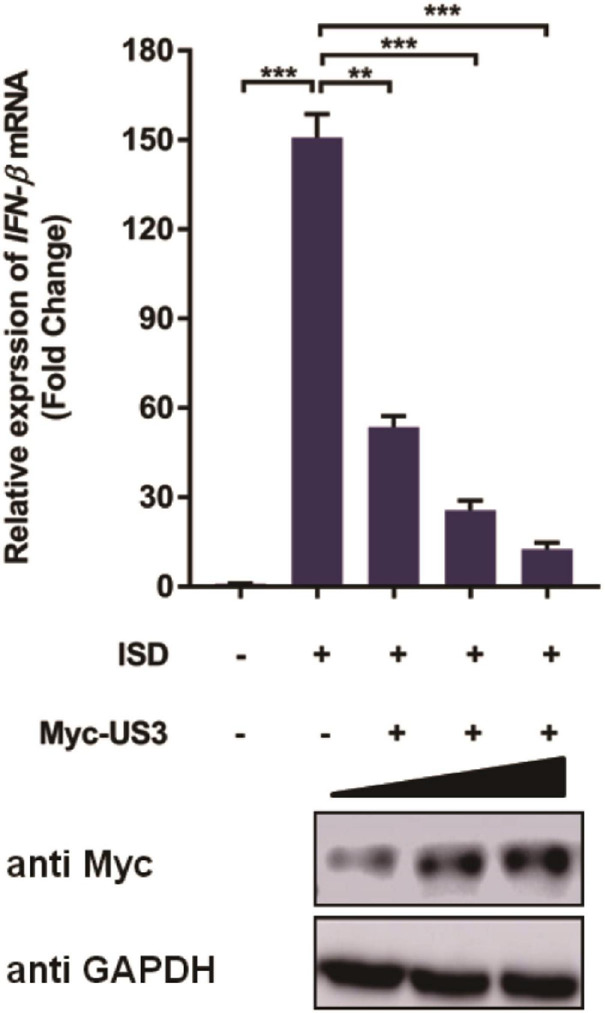
pCMV-Myc-US3 plasmid (0.2 μg, 0.5 μg, and 1.0 μg) was transfected into PK15 cells used Lipofectamine 3000. ISD (2 μg/mL) was transfected into above cells for 12 h before cells collection. US3 protein expression was detected by western blotting. An anti-Myc antibody was used and GAPDH served as the loading control. Then cellular RNA were extracted and cDNA was transcripted for *IFN-*β mRNA detection. Data were listed as mean ± SD from three independent experiments. Comparison between two groups was evaluated by unpaired Student’s *t* test. ** *p* < 0.01, *** *p* < 0.001.

Given that HSV-1 US3 can prevent IFN-β activation during infection (Wang et al., 2013; You et al., 2020), we wondered if PRV US3 protein performed a similar function via interfering with an IFN-β pathway. ISD used in this study is a double-stranded DNA 60-mer oligonucleotide derived from the HSV-1 genome. PK15 cells were transfected with a US3 expression plasmid for 24 h before ISD transfection to determine the effect of PRV US3 on IFN-β production induced by ISD. RT-qPCR results showed ISD strongly activated IFN-β mRNA expression. However, the activation was remarkably decreased in the presence of Myc-US3 expression (Figure 1), suggesting PRV US3 expression inhibits the IFN-β activation in a dose-dependent manner.

### IRF3 Might Be the Potential Target of Pseudorabies Virus US3 Protein

**FIGURE 2:**
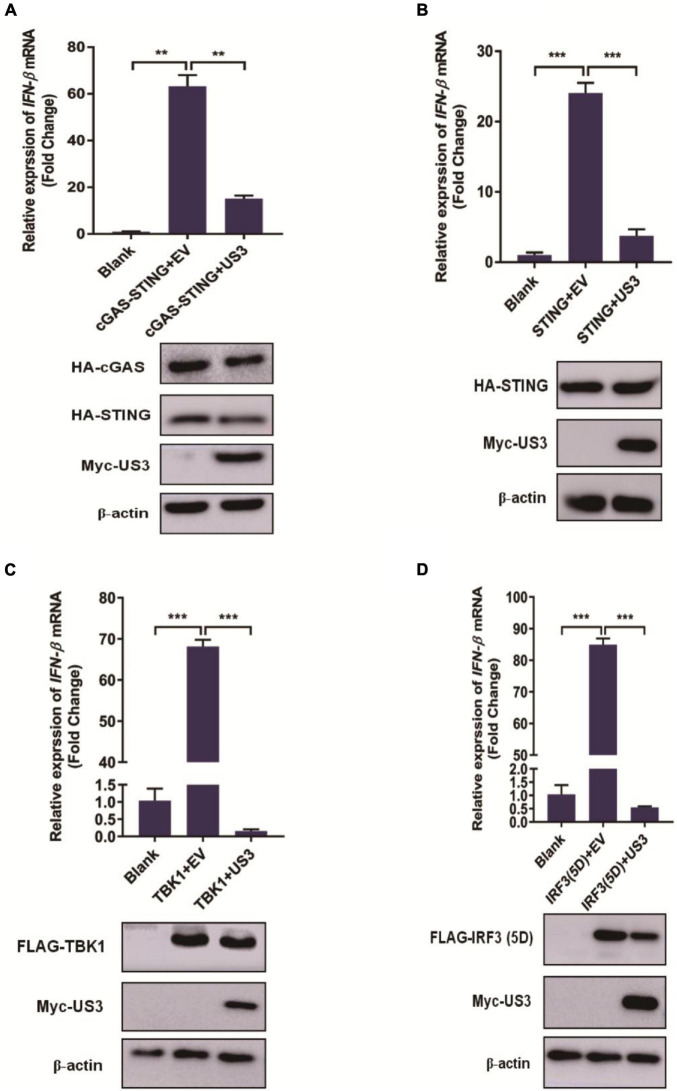
PK15 cells were cotransfected with empty vector (0.5 μg) or Myc-US3 (0.5 μg) plasmids and the indicated plasmids expressing cGAS (15 ng) + STING (2.5 ng) **(A)**, STING (0.2 μg) **(B)**, TBK1(0.2 μg) **(C)** or IRF3(5D) (0.2 μg) **(D)** for 24 h. Then cells were collected for total RNA extraction. IFN-β mRNA expression level was measured by RT-qPCR. Expression of various adaptor molecules and US3 protein was evaluated by western blotting. β-actin served as loading control. Data were listed as mean ± SD from three independent experiments. Comparison between two groups was evaluated by unpaired Student’s *t* test. ** *p* < 0.01, *** *p* < 0.001.

Type I IFN induction is mainly mediated by the cGAS-STING pathway during DNA virus infection. Cells were transfected with either plasmid encoding cGAS-STING signaling pathway adaptors TBK1, IRF3/5D, a constitutively active form of IRF3 containing five C-terminal substitutive Asp (D) residues (Ramos and Gale, 2011) and cGAS and STING proteins for 12 h before overexpressing US3 transiently for a further 24 h. Exogenous overexpression of any of these adaptor molecules significantly activated IFN-β expression. Interestingly, US3 protein expression repressed all of these adaptor molecules triggered IFN-β activation (Figures 2A–D). Thereby, we considered that IRF3 might be a targeting protein for US3 hindering the type I IFN pathway.

### US3 Interacts With IRF3 and Degrades IRF3 Through the Proteasomal Pathway

**FIGURE 3:**
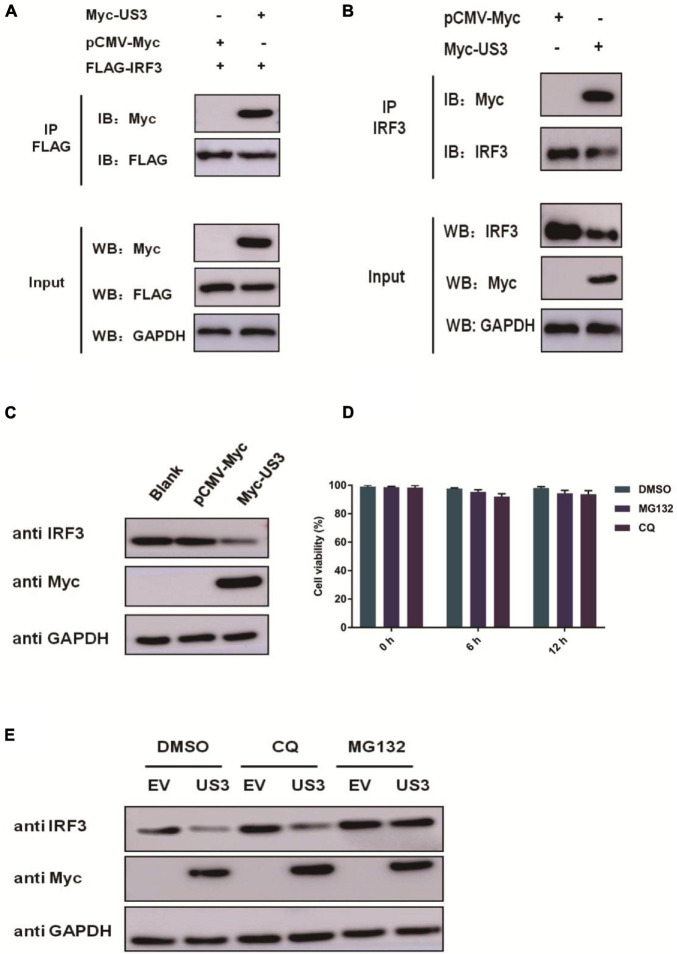
**(A)** PK15 cells were co-transfected with empty vector (0.5 μg) or Myc-US3 (0.5 μg) plasmids and FLAG-IRF3 (0.2 μg) plasmids for 30 h. The cells were then lysed and immunoprecipitated with an anti-Flag antibody. The whole-cell lysates (input) and immunoprecipitation (IP) complexes were analyzed using an anti-Myc, anti-FLAG or anti-GAPDH antibody by western blotting. **(B)** PK15 cells were transfected with empty vector (0.5 μg) or Myc-US3 (0.5 μg) plasmids for 30 h. The cells were lysed and immunoprecipitated with an anti-IRF3 antibody. The input and IP complexes were analyzed by western blotting using anti-IRF3, anti-Myc or anti-GAPDH antibodies. **(C)** PK15 cells were transfected with empty vector (0.5 μg) or Myc-US3 (0.5 μg) for 24 h, then cells were collected for western blotting using anti-IRF3, anti-Myc or anti-GAPDH antibodies. **(D)** Chemicals were previously tested for cytotoxicity at the concentrations used. PK15 cells treated with 7.5 μM MG132 or 50 μM CQ for 6 h and 12 h in 96 well plate. Cell viability was determined using the CCK8 reagent. Data were expressed as the mean ± SD from three independent experiments. **(E)** PK15 cells were transfected with Myc-US3 (0.5 μg) or empty vector (0.5 μg) for 24 h, then treated with lysosomal inhibitor MG132 (7.5 μM) or the lysosome inhibitor CQ(50 μM)for 6 h. DMSO treated cells served as vehicle control. Then cells were collected and immunoblotting for IRF3 and Myc. GAPDH served as a loading control.

IRF3 plays an extremely pivotal role in the induction of IFN in responding to viral infection. PRV US3 protein exhibited a remarkable inhibitory effect on IRF3 and its upstream adaptors (Figure 2), suggesting that it could target IRF3. PK15 cells were co-transfected with FLAG-IRF3 and Myc-US3 plasmids to test whether US3 interacted with IRF3. An anti-FLAG antibody was used to carry out co-immunoprecipitation (Co-IP) assay. As shown in Figure 3A, IRF3 coprecipitated with US3 protein, suggesting a direct interaction between US3 and IRF3 protein. To verify the interaction between US3 protein and endogenous IRF3, Co-IP was operated by transfecting PK15 cells with Myc-vector- or Myc-US3 expressing plasmids, anti-IRF3 antibody was used to detect and visualize IRF3 expression. As shown in Figure 3B, US3 protein was immunoprecipitated with endogenous IRF3.

Indeed, overexpression of US3 decreased IRF3 protein expression (Figure 3C). US3 overexpressing cells were treated with proteasome inhibitor MG132 and the autophagy inhibitor chloroquine diphosphate (CQ) to explore the mechanism by which US3 decreases IRF3 expression. We confirmed that MG132 and CQ were not toxic on PK15 cells (Figure 3D). Immunoblotting analysis revealed that MG132 inhibited IRF3 degradation but not CQ, suggesting that degradation occurs through the ubiquitination-proteasomal, not autophagic pathway (Figure 3E).

### US3 Protein Suppresses IRF3 Phosphorylation

**FIGURE 4:**
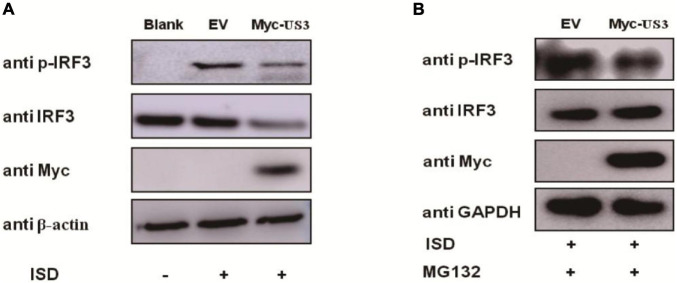
PK15 cells were transfected with empty vector (0.5 μg) or Myc-US3 (0.5 μg) plasmids for 24 h and then transfected with ISD (2 μg/mL) for 12 h. Cells were untreated **(A)** or treated **(B)** with 7.5 μM MG132 for another 6 h before collection. IRF3, phosphorylated IRF3 (p-IRF3), and US3 protein (Myc) expression was detected by immune blotting. GAPDH or β-actin served as loading control.

The phosphorylation of IRF3 is required for the induction of IFNs. Pseudorabies Virus US3 protein blocks type I IFN production by targeting IRF3. The levels of ISD-induced IRF3 phosphorylation in the absence or presence of US3 protein were examined to investigate whether US3 protein affected the phosphorylation of IRF3. PK15 cells were transfected with Myc-US3 or empty vector plasmids along with ISD. ISD induced significant phosphorylation of IRF3 in both Myc-US3 and empty vector plasmid-transfected cells. Whether treated with MG132 or not, the phosphorylation level of IRF3 was markedly lower in the Myc-US3-transfected cells than in the empty vector-transfected cells (Figures 4A,B). These results indicated that PRV US3 protein abrogated IRF3 phosphorylation.

### US3 Protein Blocks IRF3 Nuclear Translocation

**FIGURE 5:**
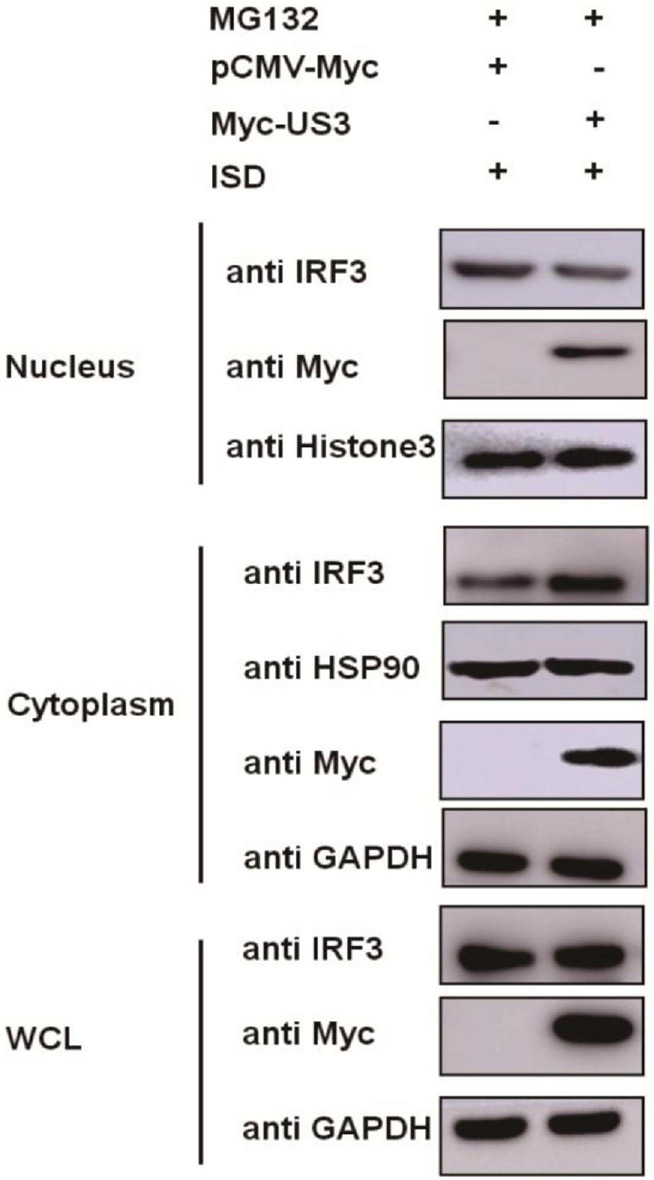
ISD (2 μg/mL) was transfected into PK15 cells in the presence of either pCMV-Myc (0.5 μg) or Myc-US3 (0.5 μg) for 24 h. Before collected, cells were treated with MG132 for 6 h. Then cytoplasmic and nuclear proteins were extracted and subjected to western blotting. Expression of IRF3 and Myc tagged US3 was detected with specific antibodies. HSP90 was used as a cytoplasmic protein marker, whereas Histone3 was used as a nuclear protein marker. GAPDH served as loading control.

IRF3 is a transcription factor that participated in type I IFN production, and its function is realized by transposition from the cytoplasm to the nucleus (Vandevenne et al., 2011). The phosphorylation of IRF3 causes its nuclear translocation. Our results showed that US3 interacts with IRF3 protein and inhibits its phosphorylation. PK15 cells were co-transfected with Myc-US3 expressing plasmids and ISD to investigate the effects of US3 protein on the nuclear translocation of IRF3. As US3 could degrade IRF3 expression through the proteasome pathway (Figure 3E), the above experimental cells were treated with MG132 for 6 h before collection. The distribution of IRF3 in cytoplasma or nucleus was detected by nuclear-cytoplasmic separation experiment. Compared with the empty vector-transfected group, in the US3 transfected group, most of the IRF3 protein remained in the cytoplasm, and only a small amount of IRF3 entered the nucleus (Figure 5). The result indicated that US3 protein inhibited ISD-induced nuclear translocation of IRF3 to prevent IFN-β production.

### Pseudorabies Virus Mutant Containing a US3 Knockdown Produces Higher Levels of Innate Immunity

**FIGURE 6:**
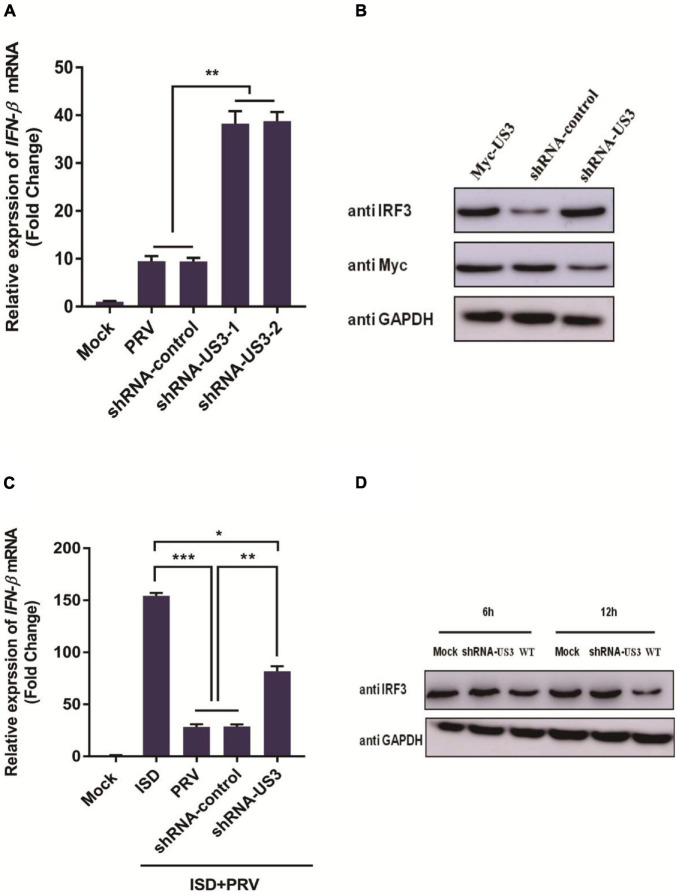
**(A)** PK15 cells were transfected with shRNA targeting US3 (0.5 μg) or control shRNA (0.5 μg) for 24 h. Then cells were infected with MOI of 1 PRV for 12 h. Cells were collected for cellular RNA extraction. cDNA was transcripted for **IFN-β** mRNA detection. Data were listed as mean ± SD from three independent experiments. Comparison between two groups were examined by unpaired Student’s *t* test. ** *p* < 0.01. **(B)** PK15 cells were transfected with shRNA-US3 (1 μg) or shRNA-control (1 μg) for 24h followed with Myc-US3 (0.5 μg) plasmid transfection. 24 h post transfection, cells were collected and lysed for IRF3 and Myc tagged US3 expression using specific antibodies. GAPDH served as loading control. **(C)** PK15 cells were transfected with shRNA-US3 (1 μg) or shRNA-control (1 μg) for 24 h followed with ISD (2 μg/mL) plasmid transfection. 12 h post transfection, above cells were uninfected or infected with MOI of 3 PRV for another 12 h. Cells were collected for total RNA extraction. 1 μg RNA was transcripted into cDNA for *IFN-*β mRNA detection. Data were expressed as mean ± SD from three independent experiments and were measured in technical duplicate. Comparisons between groups were performed by Student’s *t* test. * *p* < 0.05, ** *p* < 0.01, *** *p* < 0.001. **(D)** PK15 cells were infected with PRV Bartha strain (WT) and PRV-shRNA-US3 at 3 MOI. Endogenous IRF3 expression level was determined by western blotting at 6 h and 12 h post infection. GAPDH served as loading control.

To further determine US3 functions during a live PRV infection, two shRNAs targeted to US3 were designed and synthesized by Genechem (Shanghai, China). shRNAs targeted to US3 were transfected into PK15 cells following PRV infection. 12 h post-infection, cells were collected for RNA extraction. IFN-β mRNA expression was detected by RT-qPCR. Results showed in Figure 6A, IFN-β transcripts are present in significantly higher levels in cells transfected with shRNA targeting US3 than those transfected with shRNA-control. To verify whether this result is related to the reduced expression of US3, we examined the expression of US3 by western blotting and found that shRNA-US3 can effectively interfere with the expression of US3 (Figure 6B).

Next, we studied the effect of US3 knockdown expression on ISD-triggered IFN-β transcription. It was found that ISD can effectively stimulate the transcription of IFN-β, and PRV infection inhibited the expression of IFN-β at the mRNA level. However, in shRNA-US3 and ISD transfected cells, the inhibition of IFN-β expression by PRV was weakened (Figure 6C) but not completely disappeared, suggesting that other viral proteins may be involved in the process of resistance to host innate immunity. Altogether, these results further confirmed that US3 protein could antagonize the activation of IFN-β pathway.

To further study the expression of IRF3 protein in viral infection, PK15 cells were inoculated with 3 MOI PRV Bartha-61 strain (wild type, WT) or PRV-shRNA-US3 for 6 h and 12 h, respectively. Cells were collected for IRF3 detection. As in Figure 6D shown, levels of endogenous IRF3 in PRV-shRNA-US3 infected cells were markedly higher than in WT strain infected cells, suggesting that US3 helps to enable PRV degrade IRF3 and resistance to IFN-β signaling pathway.

## Discussion

The innate immune system composes the first line of host, and protects hosts from viral infection. The capability of viruses to avoid and regulate host innate immunity response is of great importance for viral infection (Bowie and Unterholzner, 2008). As the cGAS-MITA-TBK1 axis plays an indispensable role in host defense against DNA viruses infection (Kato et al., 2017), the DNA viruses have developed numerous means to counteract this signaling pathway for replication and latent infection (Ma and Damania, 2016). This study showed that PK15 cells infection with PRV significantly suppressed type I IFN production. We also demonstrated the role of US3 in the IFN-β signaling pathway and revealed the mechanism used by PRV to antagonize host antiviral response.

The US3 protein is a multifunctional serine/threonine-protein kinase. US3 expression modulates a wide range of cellular processes, including virus nuclear egress, inhibition of apoptosis, reorganization of the cytoskeleton, and several immune modulators (Favoreel et al., 2005; Deruelle et al., 2007; Deruelle and Favoreel, 2011). In the current study, we showed that exogenous overexpression of PRV US3 inhibited cGAS-STING, TBK1, IRF3(5D), or ISD-triggered activation of IFN-β (Figure 2). Furthermore, US3 could interact with IRF3 and degrade the protein expression level of IRF3 (Figure 3).

In this study, IRF3 was recognized as a target of PRV US3 protein, through which it inhibited type I IFN production. IRF3 is a key regulator of IFN-β pathway. It can be phosphorylated by cellular and viral proteins, conducing to either the activation or suppression of IRF3 transcriptional activities. As a consequence, leading to increase or decrease of IFN-β production. Previous studies reported that several conserved herpes virus-encoded kinases might contribute be involved in anti-IFN function by suppressing the IRF3 pathway, such as HSV-1 UL13, HCMV UL97, MHV-68 ORF36, and the EBV BGLF4 kinase protein (Hwang et al., 2009). Here, we demonstrated that PRV US3 protein antagonized the IFN-β pathway by targeting IRF3, through degradation of its protein expression, inhibits IRF3 phosphorylated and nuclear translocation (Figures 3–5). There is a direct interaction between US3 and IRF3, and endogenous IRF3 levels are affected by US3, so phosphorylated IRF3 decreased maybe result from degradation of endogenous IRF3 by US3. To rule out this effect and explore more accurately the effect of US3 on IRF3 phosphorylation, we used MG132 to treat the cells transfected with Myc-US3 and ISD or empty vector and ISD. Results found that after treated with MG132, the total IRF3 in the cells did not decrease in the Myc-US3 transfection group, but the IRF3 phosphorylation level of cells transfected with Myc-US3 was significantly lower than that of cells transfected without empty vector (Figure 4B). These results further confirmed that US3 could inhibit the phosphorylation of IRF3.

To understand the role of US3 interaction with the IFN-β signaling pathway, we used the PRV-shRNA-US3 strain for further study. When knockdown US3 expression, its ability to inhibit IFN-β transcription was weakened (Figures 6A–C). These results indicate that US3 plays an important role in antagonizing innate immunity. US3 also influences IRF3 expression. IRF3 levels in PRV-shRNA-US3-infected cells are significantly higher than those in WT strain infected cells, indicating that a virus failure to express US3 has a weakened capability to prevent IRF3 and IFN-β activation.

**FIGURE 7:**
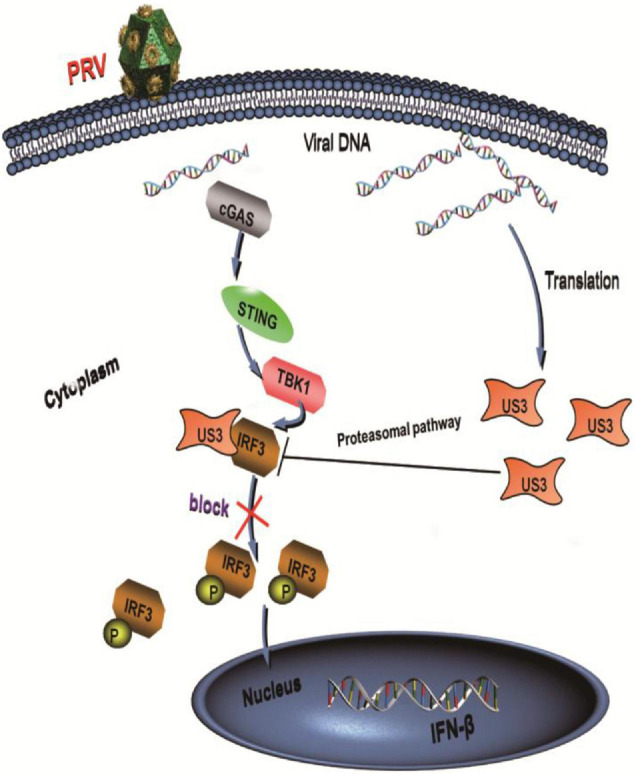
PRV protein kinase US3 interacts with IRF3 and degrades IRF3 protein expression through proteasome pathway. Additionally, US3 inhibits IRF3 phosphorylation and prevent its nuclear translocation, then negatively regulates IFN-β production.

In summary, our data demonstrated a possible mechanism that US3 antagonized IFN-β signaling pathway (Figure 7). US3 inhibited IFN-β production by targeting IRF3. There was a direct interaction between US3 and IRF3. Moreover, US3 degraded IRF3 protein level expression and blocked its activation. These findings suggested that PRV US3 could inhibit the IFN-β production and provide new insights into innate immune evasion by PRV.

## Data Availability Statement

The original contributions presented in the study are included in the article/supplementary material, further inquiries can be directed to the corresponding authors.

## Author Contributions

JX and XZ wrote the draft manuscript. JX and RF designed the experiment. JX, XZ, LC, YB, SX, and XL performed experiments and processed the data. AI, YZ, and RF revised and proofread the draft manuscript. RF supervised the entire process. All authors contributed to the article and approved the submitted version.

## Conflict of Interest

The authors declare that the research was conducted in the absence of any commercial or financial relationships that could be construed as a potential conflict of interest.

## Publisher’s Note

All claims expressed in this article are solely those of the authors and do not necessarily represent those of their affiliated organizations, or those of the publisher, the editors and the reviewers. Any product that may be evaluated in this article, or claim that may be made by its manufacturer, is not guaranteed or endorsed by the publisher.
